# Common vitamin D pathway gene variants reveal contrasting effects on serum vitamin D levels in African Americans and European Americans

**DOI:** 10.1007/s00439-014-1472-y

**Published:** 2014-08-02

**Authors:** Ken Batai, Adam B. Murphy, Ebony Shah, Maria Ruden, Jennifer Newsome, Sara Agate, Michael A. Dixon, Hua Yun Chen, Leslie A. Deane, Courtney M. P. Hollowell, Chiledum Ahaghotu, Rick A. Kittles

**Affiliations:** 1 Division of Urology, Department of Surgery, University of Arizona College of Medicine, University of Arizona Cancer Center, P.O. Box 245024, 1515 N. Campbell Ave., Tucson, AZ 85724 USA; 2 Department of Urology, Feinberg School of Medicine, Northwestern University, Chicago, USA; 3 Jesse Brown Veterans Affairs Medical Center, Chicago, USA; 4 Department of Medicine, University of Illinois at Chicago, Chicago, USA; 5 Center for Clinical and Translational Science, University of Illinois at Chicago, Chicago, USA; 6 Division of Health Policy and Administration, School of Public Health, University of Illinois at Chicago, Chicago, USA; 7 Division of Epidemiology and Biostatistics, School of Public Health, University of Illinois at Chicago, Chicago, USA; 8 Department of Urology, Rush University Medical Center, Chicago, USA; 9 Section of Urology, Department of Surgery, Cook County Health and Hospitals System, Chicago, USA; 10 Division of Urology, Department of Surgery, Howard University Hospital, Washington, D.C., USA

## Abstract

Vitamin D deficiency is more common among African Americans (AAs) than among European Americans (EAs), and epidemiologic evidence links vitamin D status to many health outcomes. Two genome-wide association studies (GWAS) in European populations identified vitamin D pathway gene single-nucleotide polymorphisms (SNPs) associated with serum vitamin D [25(OH)D] levels, but a few of these SNPs have been replicated in AAs. Here, we investigated the associations of 39 SNPs in vitamin D pathway genes, including 19 GWAS-identified SNPs, with serum 25(OH)D concentrations in 652 AAs and 405 EAs. Linear and logistic regression analyses were performed adjusting for relevant environmental and biological factors. The pattern of SNP associations was distinct between AAs and EAs. In AAs, six GWAS-identified SNPs in *GC*, *CYP2R1*, and *DHCR7/NADSYN1* were replicated, while nine GWAS SNPs in *GC* and *CYP2R1* were replicated in EAs. A *CYP2R1* SNP, rs12794714, exhibited the strongest signal of association in AAs. In EAs, however, a different *CYP2R1* SNP, rs1993116, was the most strongly associated. Our models, which take into account genetic and environmental variables, accounted for 20 and 28 % of the variance in serum vitamin D levels in AAs and EAs, respectively.

**Electronic supplementary material:**

The online version of this article (doi:10.1007/s00439-014-1472-y) contains supplementary material, which is available to authorized users.

## Introduction

Many chronic diseases are disproportionately distributed in the US populations. Diabetes mellitus, cardiovascular diseases, several malignancies, and other diseases are more prevalent among African Americans (AAs) relative to European Americans (EAs) and Asian Americans. Differences in serum vitamin D [25(OH)D] concentration among racial/ethnic groups are suspected to be one of the sources of health disparities (Grant and Peiris 2010; Harris 2011). Numerous studies demonstrated that AAs have significantly lower serum 25(OH)D levels than EAs (Benjamin et al. 2009; Chan et al. 2010; Ginde et al. 2009; Harris et al. 2000; Looker et al. 2008; Murphy et al. 2012; Nesby-O’Dell et al. 2002; Shea et al. 2011; Tseng et al. 2009). Vitamin D deficiency is common even among AAs who live in sunlight intense southern and southwestern states or who have higher dietary vitamin D intake than the longstanding recommended daily allowance (≥400 IU/day) (Egan et al. 2008; Jacobs et al. 2008; Tseng et al. 2009). Although the causal mechanism involved in risk is unknown, there is also epidemiologic evidence linking vitamin D status to several types of cancer (e.g., breast, colon, and prostate cancer), bone diseases (e.g., rickets, osteomalacia, and osteoporosis), cardiovascular diseases, diabetes, autoimmune diseases, microbial infections, childhood asthma and allergy, and other health conditions (Grant and Peiris 2010, 2012; Hossein-nezhad and Holick 2013; Litonjua 2012). High disease burden in AAs could be due to the contrasting effects of dark skin pigmentation which evolved in their ancestral African environment and low ultraviolet radiation (UVR) in their new environment (Jablonski and Chaplin 2012).

Circulating levels of 25(OH)D are strongly influenced by multiple factors. Various studies have demonstrated that dietary intake, dietary supplement use, season of blood draw, UVR exposure, smoking, sex, age, body mass index (BMI), and race/ethnicity are important predictors of serum 25(OH)D levels (Chan et al. 2010; Egan et al. 2008; Murphy et al. 2012; Shea et al. 2011). Of these predictors, many studies repeatedly demonstrated the strong associations of vitamin D intake and season of blood draw with serum 25(OH)D levels.

Genetic epidemiological studies also identified variants that were associated with serum 25(OH)D levels. Two meta-analyses of genome-wide association studies (GWAS) in European descent populations found single-nucleotide polymorphisms (SNPs) in vitamin D pathway genes associated with serum 25(OH)D levels (Ahn et al. 2010; Wang et al. 2010). In these GWAS meta-analyses, the strongest signals of association were observed in *GC* (vitamin D binding protein), *DHCR7* (7-dehydrocholesterol reductase) and *NADSYN1* region, and *CYP2R1* (cytochrome P450, family 2, subfamily R, polypeptide 1). Smaller scale replication and candidate gene studies in European and Asian populations also demonstrated the association of variants in these gene regions with serum 25(OH)D levels (Bu et al. 2010; Cooper et al. 2011; Engelman et al. 2013; Lu et al. 2012; Zhang et al. 2012). On the other hand, in AAs, studies have shown that genetic ancestry contributes to serum 25(OH)D variation (Signorello et al. 2010; Yao et al. 2012), but the association of the GWAS-identified SNPs with serum 25(OH)D levels has not been fully explored. Only a few GWAS-identified variants, located in *GC*, show significant association with serum 25(OH)D levels in AAs (Engelman et al. 2008; Powe et al. 2013; Signorello et al. 2011). Here, we investigated if 39 SNPs in eight vitamin D pathway genes were associated with serum 25(OH)D concentrations in AAs and EAs.

## Materials and methods

### Subjects

A total of 652 AA men (226 AAs from Washington, D.C. and 426 AAs from Chicago) and 405 EA men from Chicago were included for this study. The subjects from Washington, D.C. were recruited at Howard University Hospital (Bonilla et al. 2011; Robbins et al. 2011). The participants from Chicago were recruited at University of Illinois Hospital and Health Sciences System, Northwestern Memorial Hospital, Cook County Health and Hospital System, Jesse Brown Veterans Affairs Medical Center, and University of Chicago Hospital (Murphy et al. 2012). All of the participants were unrelated and self-identified as AA or EA. Individuals with liver and/or chronic kidney disease were excluded from the analyses.

Blood samples for 25(OH)D assays and DNA analysis, demographic information, and information on potential modifiers of serum 25(OH)D were collected at the time of recruitment. Research coordinators conducted in-person interviews, administered structured questionnaires, and obtained information on ancestry, medical history, income, education, marital status, and lifetime history of sun exposure (Murphy et al. 2012). Skin color of upper inner arm was measured using a portable narrow-band reflectometer, called DermaSpectrometer (Cyberderm, PA) (Shriver and Parra 2000; Shriver et al. 2003). Dietary vitamin D intake was assessed using a Block calcium and vitamin D screener validated for use in the AA population (Block et al. 1990; Coates et al. 1991). UVR exposure was assessed using a questionnaire that evaluates outdoor activities and geographic residence. The serum samples were stored at −20 °C until 25(OH)D measurement. Total 25(OH)D concentration was assessed using the Diasorin^®^ chemiluminescence immunoassay method in the Department of Pathology NorthShore University HealthSystem. We defined vitamin D deficiency as serum 25(OH)D levels <50 nmol/l (<20 ng/ml) and vitamin D insufficiency as 25(OH)D levels between 50 and 75 nmol/l (20 and 30 ng/ml) (Holick 2007; Holick et al. 2011).

### Genetic analysis

We genotyped 39 SNPs in eight vitamin D metabolic pathway genes (*GC*, *DHCR7/NADSYN1*, *VDR*, *CYP2R1*, *CYP27A1*, *CYP27B1*, *CYP3A4*, and *CYP24A1*), including 19 GWAS-identified variants (Table 1; Supplementary Table 1) (Ahn et al. 2010; Wang et al. 2010). SNP selection process was previously described in Pibiri et al. (2014). Individual genetic ancestry was determined for each person using 105 autosomal DNA ancestry informative markers (AIMs) for West African, Native American, and European genetic ancestry using published methods (Giri et al. 2009; Tian et al. 2006). All the genotyping was performed using iPLEX Sequenome MassARRAY. All the individuals included in the study had genotyping calling rate of >95 %.

**Table 1:** Vitamin D pathway genes investigated

Chromosome	Gene	Number of SNPs	Full name of gene
2	*CYP27A1*	1	Cytochrome P450, family 27, subfamily A, polypeptide 1
4	*GC*	9	Vitamin D binding protein
7	*CYP3A4*	1	Cytochrome P450, family 3, subfamily A, polypeptide 4
11	*CYP2R1*	5	Cytochrome P450, family 2, subfamily R, polypeptide 1
	*DHCR7/NADSYN1*	8	7-Dehydrocholesterol reductase/NAD synthetase 1
12	*VDR*	7	Vitamin D receptor
	*CYP27B1*	2	Cytochrome P450, family 27, subfamily B, polypeptide 1
20	*CYP24A1*	6	Cytochrome P450, family 24, subfamily A, polypeptide 1

### Statistical analysis

Allelic association tests were performed using linear regression models to investigate the association with log-transformed serum 25(OH)D levels and logistic regression models to test the association with vitamin D deficiency (<50 nmol/l). We performed separate analyses for AAs and EAs because of differences in linkage disequilibrium (LD), allele frequencies, and biological and environmental factors contributing to serum 25(OH)D levels in the two populations. For the analyses of AAs, we adjusted for age, West African ancestry (WAA), and study site (Model 1, *n* = 652). We also tested for association adjusting for age, WAA, study site, and environmental predictors (Model 2, *n* = 557). We adjusted for WAA to control for population stratification in the admixed AA populations. For the analyses of EAs, we adjusted for age (Model 1, *n* = 405), and we performed analyses further adjusting for environmental predictors (Model 2, *n* = 385). We treated total vitamin D intake, season of blood draw, and UVR exposure as binary variables. We treated total vitamin D intake as a binary variable (<400 IU/day vs. ≥400 IU/day, Institute of Medicine Estimated Average Requirement), because it was not normally distributed. We defined from June to November as UVR high months and from December to May as low UVR months to account for serum 25(OH)D decay time. Mean serum 25(OH)D levels were not different between individuals with UVR medium and low exposure, so the levels of UVR exposure were categorized into high vs. low/medium exposure. For GWAS-identified SNPs, we considered the *P* < 0.05 as the threshold for statistical significance. For other SNPs, pointwise empirical *P* values and empirical *P* values corrected for multiple testing were obtained using the max(*T*) permutation procedure (10,000 permutations) using PLINK (Purcell et al. 2007). Additional statistical analyses were conducted using IBM SPSS Statistics, version 21.0 (IBM Corp., Armonk, NY). We derived a Genetic Risk Score for vitamin D deficiency by adding the number of risk alleles of the top two most significantly associated SNPs in AAs and EAs. Power analysis was performed using SPSS SamplePower (IBM Corp., Armonk, NY).

Genetic ancestry was estimated using STRUCTURE software from the AIMs genotyped (Falush et al. 2003; Pritchard et al. 2000). We ran STRUCTURE under the admixture model using prior population information and independent allele frequencies with Markov Chain Monte Carlo (MCMC) method using *K* = 3 parental populations (West African, European, and Native American) and a burn-in length of 30,000 for 70,000 repetitions. Each participant was scored from 0 to 100 % for individual estimates of West African, Native American and European ancestry. Mean WAA in AAs was 0.79, which was similar to previous estimates in the study populations (Batai et al. 2012). LD patterns were examined using HaploView (Barrett et al. 2005). Synthesis-View was used to visualize the results of SNP associations in linear regression models (Pendergrass et al. 2010).

## Results

Vitamin D deficiency and insufficiency were more common in AAs than in EAs (Table 2; Supplementary Fig. 1). The majority of our AA study participants (85.8 %) were vitamin D insufficient or deficient with 25(OH)D levels <75 nmol/l. Although many of them were recruited in the high UV months, more than half of them (57.5 %) were vitamin D deficient with 25(OH)D levels <50 nmol/l. Mean serum 25(OH)D levels was significantly higher in EAs than in AAs, and only 28.9 % of EA study participants were vitamin D deficient. Total vitamin D intake was lower in AAs than in EAs, and significantly higher proportion of EAs had total vitamin D intake ≥400 IU/day (*P* < 0.001). A much smaller proportion of AAs (14.9 %) had the Institute of Medicine Recommended Dietary Allowance (≥600 IU/day) compared to EAs (25.4 %).

**Table 2:** Study participants’ characteristics

	African Americans (*n* = 652)	European Americans (*n* = 405)	*P* values^a^
Age, mean (SD)	59.0 (10.0)	60.9 (8.4)	0.001
25(OH)D (nmol/l), mean (SD)	47.8 (24.2)	64.9 (28.2)	<0.001
Vitamin D status^b^, %			<0.001
Severe deficient	14.1	4.2	
Deficient	43.4	24.7	
Insufficient	28.3	38.8	
Sufficient	13.2	32.4	
UV high season, from June to November, %	62.1	39.0	<0.001
Total vitamin D intake ≥ 400 IU/day, %	36.4	49.7	<0.001

^a^ *P* values were calculated from independent sample *T* test for continuous variables and *χ* ^2^ test for categorical variables

^b^Vitamin D severe deficient (<25.0 nmol/l), deficient (25.0–49.9 nmol/l), insufficient (50.0–74.9 nmol/l), and sufficient (≥75.0 nmol/l)

Before we tested SNP associations, we performed multiple linear regression analyses to identify biological and environmental modifiers of serum 25(OH)D concentrations (Supplementary Table 2). Among AAs, study site, total vitamin D intake and season of blood draw were significantly associated with serum 25(OH)D levels (*P* < 0.001). Skin pigmentation and WAA were not associated with serum 25(OH)D levels. In EAs, age (*P* = 0.006), season of blood draw (*P* < 0.001), total vitamin D intake (*P* < 0.001), and body mass index (BMI) (*P* < 0.001) were significantly associated with serum 25(OH)D levels. Also, UVR exposure was marginally associated with serum 25(OH)D levels (*P* = 0.06) in EAs. These significant modifiers of serum 25(OH)D levels were included in the linear and logistic regression models in our genetic association analyses.

Of the 39 SNPs genotyped, two SNPs in *VDR*, rs2228570 (*Fok*I) and rs1989969, were hypervariable loci exhibiting more than two alleles, and we removed them from our analyses. We also excluded rs11568820 (*Cdx*2) from the analyses in AAs, because it deviated from Hardy–Weinberg Equilibrium (*P* < 0.001). A *CYP27A1* SNP, rs116071925, was excluded in the analyses of EAs, because it was monomorphic.

**Fig. 1:**
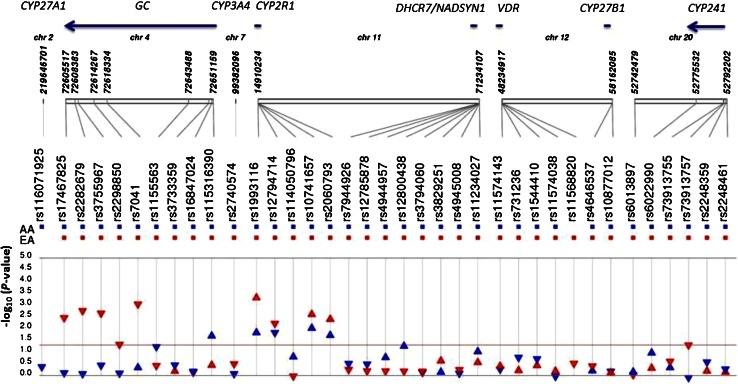
−log_10_ *P* value from linear regression analysis (Model 2) in African Americans (*Blue*) and European Americans (*Red*). Additive effect of minor allele was tested, and the *triangles* indicate direction of effect (*β*). The *horizontal line* shows the statistical significance threshold at *α* = 0.05

In the linear regression models among AAs, we observed stronger associations of SNPs with serum 25(OH)D levels when we additionally adjusted for vitamin D intake and season of blood draw (Model 2) than when only age, WAA, and study site were included (Model 1). In Model 1, three GWAS-identified SNPs (1 in *CYP2R1* and 2 in *DHCR7/NADSYN1*) were significantly associated with serum 25(OH)D levels (Supplementary Table 3). Although the number of individuals included was smaller in Model 2 than in Model 1, five GWAS-identified SNPs (1 in *GC* and 4 in *CYP2R1*) and one non-GWAS-identified SNP in *GC* were significantly associated with serum 25(OH)D levels in Model 2 (Fig. 1; Table 3). Two *CYP2R1* SNPs, rs12794714 and rs10741657, equally showed strong association (*P* = 0.01). These two SNPs were weakly linked (*r* ^*2*^ = 0.07) (Supplementary Fig. 2). When these two SNPs were included in the regression model, they showed independent association (*P* = 0.04 for both rs10741657 and rs12794714). The logistic regression analysis for vitamin D deficiency supports the strong associations of *CYP2R1* SNPs, and rs12794714 showed the strongest association with vitamin D deficiency (*P* = 0.003, OR = 1.72, 95 % C.I.; 1.20–2.47). A previously reported *GC* SNP, rs115563, was weakly associated with serum 25(OH)D levels (*P* = 0.048). Although not significant after correcting for multiple testing, another SNP in *GC*, rs115316390, that was not reported in the GWAS meta-analyses showed stronger association with serum 25(OH)D levels. This SNP was not in LD with rs1155563 (*r* ^*2*^ < 0.001), and they were independently associated (*P* = 0.03 for both rs1155563 and rs115316390), when they were adjusted for each other in the regression model. Although the two SNPs in *DHCR7/NADSYN1* were not associated with 25OHD in Model 2 in the linear regression analysis, rs12800438 was significantly associated with vitamin D deficiency (*P* = 0.04, OR = 0.76, 95 % C.I.; 0.58–0.99) in AAs.

**Table 3:** SNPs associated with serum 25(OH)D levels in African Americans and European Americans

Chromosome	Gene	SNPs	Position	African Americans^a^				European Americans^b^			
			(BP)^c^	MA^d^	*β*	*P* values	*P*_EMP_^e^	MA^d^	*β*	*P* values	*P*_EMP_^e^
4	*GC*	rs17467825	72605517	G	−0.01	0.62		G	−0.04	**0.003**	
		rs2282679	72608383	C	−0.01	0.68		C	−0.05	**0.001**	
		rs3755967	72609398	A	−0.02	0.30		A	−0.04	**0.002**	
		rs2298850	72614267	G	−0.01	0.65		G	−0.03	**0.04**	
		rs7041	72618334	G	0.01	0.57		T	−0.04	**0.0007**	
		rs1155563	72643488	C	−0.04	**0.048**		C	−0.02	0.31	
		rs115316390	72651159	A	0.17	**0.03**	0.33	A	0.07	0.44	1.00
11	*CYP2R1*	rs1993116	14910234	T	0.03	**0.02**		T	0.04	**0.0006**	
		rs12794714	14913575	A	−0.04	**0.01**		A	−0.04	**0.005**	
		rs10741657	14914878	A	0.04	**0.01**		A	0.04	**0.003**	
		rs2060793	14915310	A	0.03	**0.02**		A	0.04	**0.005**	
20	*CYP24A1*	rs73913757	52790518	T	0.00	0.99	1.00	T	−0.09	**0.04**	0.49

Statistically significant *P* values at α = 0.05 are bolded

^a^Adjusted for age, WAA, study site, total vitamin D intake, and season of blood draw (Model 2)

^b^Adjusted for age, total vitamin D intake, season of blood draw, BMI, and UVR exposure (Model 2)

^c^Base pair position on the chromosome is based on GRCh37/hg19

^d^Minor Allele

^e^Empirical *P* values obtained using max(*T*) permutation procedure (10,000 permutations) correcting for multiple testing. Permutation test was not performed for GWAS-identified SNPs

A contrasting pattern of associations was observed in EAs. Compared to AAs, there were more SNPs with stronger signal of association in both Models (Fig. 1; Supplementary Table 4), and different SNPs from those found in AAs showed stronger association. Nine GWAS-identified SNPs were significantly associated with serum 25(OH)D concentrations. A *CYP2R1* SNP, rs1993116, showed the strongest evidence of association (*P* = 0.0006). After adjusting for rs1993116, no other SNP in *CYP2R1* remained significant. The logistic regression analysis confirmed the strongest association of this SNP (*P* = 0.0008, OR = 0.51, 95 % C.I.; 0.35–0.76). A *GC* SNP, rs7041, showed the second strongest association (*P* = 0.0007), and after conditioning on this SNP in the regression analyses, other *GC* SNPs were no longer significantly associated with serum 25(OH)D levels. Figure 1 shows the different directions of association (*β*) in AAs and EAs for rs7041. However, different alleles were tested in AAs and EAs, because the minor allele in AAs was the major allele in EAs, and rs7041 was not associated with serum vitamin D levels in AAs. In addition, a *CYP24A1* SNP, rs73913757, was associated with serum 25(OH)D levels (*P* = 0.04), but it was not significant after controlling for multiple testing.

**Fig. 2:**
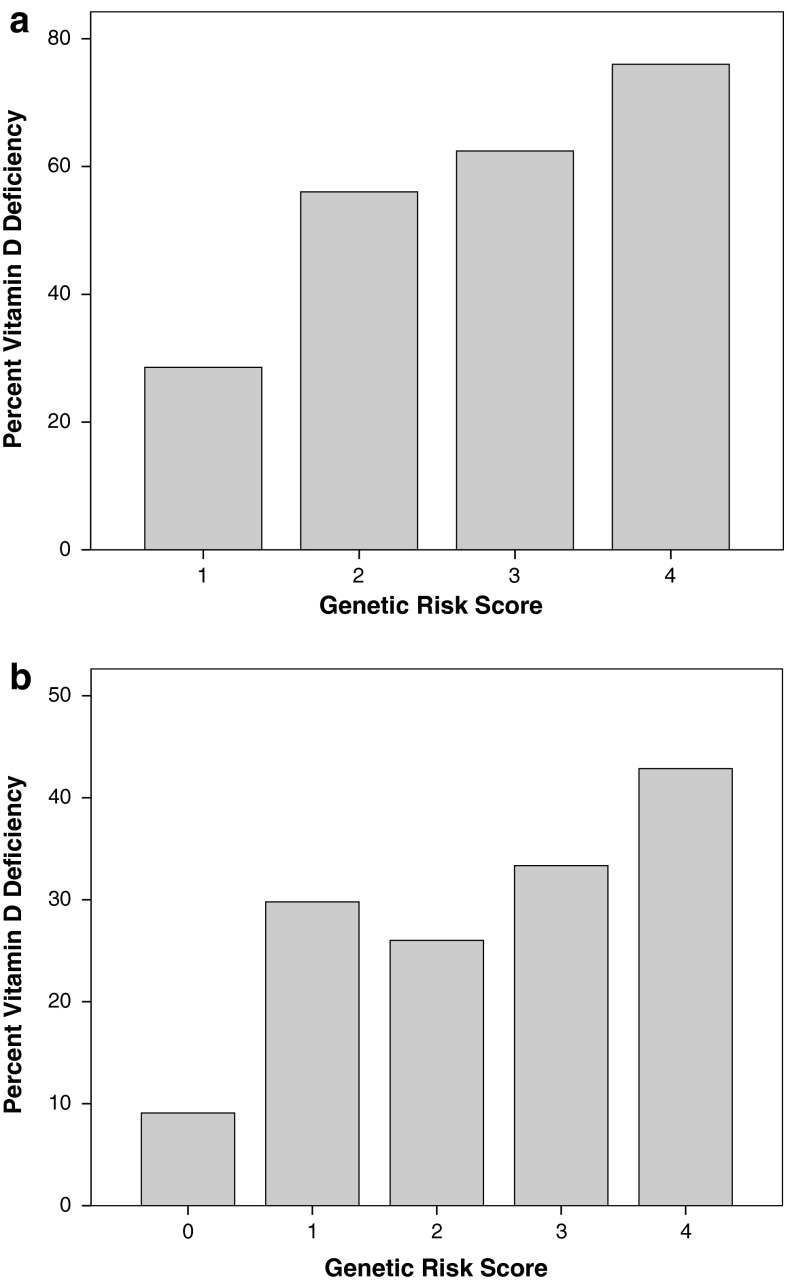
Percent vitamin D deficiency for each unweighted Genetic Risk Score using top two SNPs; rs12794714 (*CYP2R1*) and rs115316390 (*GC*) in African Americans (**a**), and rs1993116 (*CYP2R1*) and rs7041 (*GC*) in European Americans (**b**)

To understand the additive effects of the significant vitamin D pathway gene variants on serum 25(OH)D levels, we performed linear regression analysis by adding the top two independently associated SNPs to the linear regression model with the biological and environmental modifiers. In AAs, the model including age, WAA, study site, total vitamin D intake, and season of blood draw explained 19.1 % of the variance in serum 25(OH)D levels (adjusted *R* ^*2*^ = 0.191). We added a *CYP2R1* SNP, rs12794714, that showed the strongest association in single SNP linear regression and logistic regression analysis, and a *GC* SNP, rs115316390, to the linear regression model. The two SNPs together explained an additional 1.1 % of the serum 25(OH)D variation (adjusted *R* ^*2*^ = 0.202). Genetic Risk Score was calculated using the top two SNPs by adding the number of A alleles for rs12794714 (*CYP2R1*) and G alleles for rs115316390 (*GC*). The Genetic Risk Score ranged from one to four (Fig. 2a). As the Genetic Risk Score increased, proportion of vitamin D-deficient individuals increased. A small proportion (28.6 %) of AAs who carry one risk allele was vitamin D deficient. Vitamin D deficiency was very common in AAs who carry four risk alleles (76.0 %). In linear regression model adjusting for age, WAA, study site, season, and vitamin D intake, the Genetic Risk Score was significantly associated with serum 25(OH)D levels (*β* = −0.044, *P* = 0.005). The regression coefficient (*β*) estimates for these two SNP were different, so we weighted the Genetic Risk Score using *β* estimates. In the same linear regression model, the weighted Genetic Risk Score was also significantly associated with serum 25(OH)D levels (*β* = −0.947, *P* = 0.001).

Similarly, compared to biological and environmental modifiers, genetic variation explained a small proportion of serum 25(OH)D variation in EAs. SNPs and biological and environmental modifiers, however, accounted for greater serum 25(OH)D variation in EAs than in AAs. Adjusted *R* ^*2*^ in the linear regression model including the age, total vitamin D intake, season of blood draw, BMI, and UVR exposure was 0.241. The model including two SNPs, rs7041 (*GC*) and rs1993116 (*CYP2R1*) and environmental variables accounted for 28.2 % of variance (adjusted *R* ^*2*^ = 0.282). Genetic Risk Score was calculated with sum of the number of C alleles for rs1993116 (*CYP2R1*) and T alleles for rs7041 (*GC*). A very small proportion (9.1 %) of EAs who had zero risk allele was vitamin D deficient (Fig. 2b). Vitamin D deficiency was less common in EAs than in AAS, and less than half (42.9 %) of EAs who had four risk alleles were vitamin D deficient. The Genetic Risk Score was also strongly associated with serum 25(OH)D levels (*β* = 0.043, *P* < 0.001) in linear regression model adjusting for age, BMI, season, vitamin D intake, and UV exposure. Because the regression coefficient was virtually identical for two SNPs, we did not perform analysis with weighted Genetic Risk Score.

Increments of *R* ^*2*^ for rs12794714 and rs115316390, when they were added to the regression model (Model 2) in AAs, were 0.008 and 0.006, respectively. Our AA samples provide only 65 and 56 % power for these two SNPs to detect the effect size at a significance level of *α* = 0.05. In EAs, increments of *R* ^*2*^ for the top two SNPs were larger. When rs7041 and rs1993116 were added to the regression models with the other modifiers, *R* ^*2*^ increase were 0.022 and 0.021, respectively. Our EA samples have 90 and 89 % power to detect the observed *R* ^*2*^ increment.

## Discussion

In this study of 652 AAs and 405 EAs, we investigated the association of 37 candidate SNPs in eight vitamin D pathway gene regions, and we successfully replicated GWAS findings in our AA and EA populations demonstrating that six SNPs in three vitamin D pathway genes in AAs and nine SNPs in two genes in EAs were significantly associated with serum 25(OH)D levels or vitamin D deficiency (Supplementary Table 5). We also found a previously unreported SNP in *GC* among AAs and a SNP in *CYP24A1* among EAs that were associated with serum 25(OH)D levels, though they were not significantly associated after correcting for multiple testing. The strength of associations and the SNPs that showed the strongest signal of association were, however, different between AAs and EAs.

When human skin is exposed to UVR, 7-dehydrocholesterol is converted to pre-vitamin D_3_ (Holick 2007). Also within the skin, the enzyme, 7-dehydrocholesterol reductase (DHCR7) catalyzes the conversion of 7-dehydrocholesterol to cholesterol. Thus, increased activity of DHCR7 potentially lowers the bioavailability of 7-dehydrocholesterol for vitamin D synthesis. Two GWAS meta-analyses identified SNPs in *DHCR7* and a nearby gene, *NADSYN1*, strongly associated with serum 25(OH)D levels in European populations (Ahn et al. 2010; Wang et al. 2010). We, however, failed to replicate these findings in our EA samples. In our AA sample, one SNP in this region was weakly associated with vitamin D deficiency (*P* = 0.04). Decreased ability to synthesize vitamin D due to older age and/or darker skin pigmentation may explain the inconsistent results between our study and the two GWAS meta-analyses (Armas et al. 2007; Clemens et al. 1982; MacLaughlin and Holick 1985). Our study participants are older individuals living in urban areas at northern latitudes where high UVR is available for only several months of the year (Fioletov et al. 2010). Also, in our study participants, skin pigmentation was not associated with serum 25(OH)D levels. A major source of vitamin D among them appears to be dietary intake rather than UVR, and in the linear regression analysis, vitamin D dietary intake explained a higher proportion of serum vitamin D variance than season of blood draw or UVR. Nonetheless, this is the first study to investigate the association of *DHCR7/NADSYN1* SNPs with serum 25(OH)D levels among people of African descent.

Vitamin D that is synthesized in the skin or consumed from the diet is converted to a circulating form of vitamin D, 25(OH)D, in the liver with the enzyme, hydroxylase, and DNA sequence variants in these hydroxylase genes, such as *CYP2R1*, may impact vitamin D metabolism. In our study, SNPs in *CYP2R1* showed the strongest signal of association in both AAs and EAs. The *CYP2R1* SNP, rs1993116, was most strongly associated with serum vitamin D status in our EA subjects and also showed the strongest signal of association within this gene in one of the GWAS meta-analyses (Ahn et al. 2010). However, rs12794714, the *CYP2R1* SNP that consistently showed the strongest association with serum 25(OH)D levels and vitamin D deficiency in our AA subjects, was not the most strongly associated SNP in either GWAS meta-analyses. Two other studies investigated the association of *CYP2R1* SNPs and serum 25(OH)D levels in AAs, but none of the associations were significant (Pillai et al. 2011; Signorello et al. 2011). The different associations observed for these two *CYP2R1* SNPs between AA and EA populations maybe due to differences in LD across populations. While rs1993116 and rs1279714 were moderately linked in EAs (*r* ^*2*^ = 0.46), the two SNPs show weak linkage in AAs (*r* ^*2*^ = 0.05) (Supplementary Fig. 2). The LD difference suggests that a functional variant exists in the vicinity of these SNPs.

After vitamin D is converted to 25(OH)D, 25(OH)D binds to the vitamin D binding protein and is transported to target tissues through the circulation system. Sequence variants may reduce expression of *GC* or ability of vitamin D binding protein to bind to 25(OH)D. Thus, SNPs within *GC* may reduce circulating 25(OH)D levels. The two GWAS meta-analyses found the strongest signal of association with SNPs in *GC* (Ahn et al. 2010; Wang et al. 2010). In these GWAS, rs2282679 had the lowest *P* value, but other *GC* SNPs showed stronger associations than rs2282679 in our AA and EA populations. Although the association of rs2282679 has been consistently replicated in many smaller scale replication and candidate gene studies in African Americans, European Americans, and Asians, these studies often find other SNPs in *GC* showing stronger association than the GWAS top hit (Bu et al. 2010; Lu et al. 2012; Signorello et al. 2011; Zhang et al. 2012, 2013).

The SNP, rs7041, in exon 11 of *GC* creates an Asp → Glu amino acid change in the vitamin D binding protein. In our EA population, rs7041 showed the strongest association with serum 25(OH)D levels. The association of this SNP with 25(OH)D levels was consistently demonstrated in the two GWAS meta-analyses (Ahn et al. 2010; Wang et al. 2010) and in smaller studies in European populations (Cooper et al. 2011; Engelman et al. 2013; Sinotte et al. 2009) as well as in AAs (Engelman et al. 2008; Powe et al. 2013). Along with this SNP, another missense mutation at rs4588 (Thr → Lys) was associated with serum 25(OH)D and vitamin D binding protein levels in AAs and other racial/ethnic groups, demonstrating the functional importance of these two SNPs (Carpenter et al. 2013; Powe et al. 2013). We note that the minor allele of rs7041 (G) in our AAs was the major allele in EAs, and this allele was positively correlated with serum 25(OH)D in our EAs, but not in our AA samples or in another study in AAs (Signorello et al. 2011). Instead, Signorello et al. (2011) found the strongest association with rs2298849, which is less than 1 kb away from rs1155563, the SNP that was significantly associated with 25(OH)D levels in our AAs. These two *GC* SNPs exhibited very little LD in African descent populations (*r* ^*2*^ = 0.03 in YRI and *r* ^*2*^ = 0.04 in ASW). The SNP, rs115316390, located in the intron region showed the strongest *GC* association in our AA population (*P* = 0.03). Although the association was not significant after correcting for multiple testing, this study is the first to report the evidence of association of rs11531690 with serum 25(OH)D concentrations. These data suggest that there could be other functional variants in the vicinity of these SNPs affecting serum 25(OH)D and vitamin D binding protein levels. It should also be noted that while serum vitamin D binding protein levels stay relatively stable over time, serum 25(OH)D levels can seasonally fluctuate (Harris and Dawson-Hughes 1998; Sonderman et al. 2012).

Contrasting patterns between AAs and EAs were also observed when significantly associated SNPs in *CYP2R1* and *GC* were added to our full linear regression model with the relevant covariates. Two SNPs, one each from *CYP2R1* and *GC*, together explained more of the variation in serum 25(OH)D levels in EAs than in AAs (4.1 % increase in *R* ^*2*^ among EAs compared to a 1.1 % increase among AAs). Nevertheless, these SNPs account for a very small proportion of variation in serum 25(OH)D levels in both populations compared to behavioral, biological, and environmental predictors.

Several studies have shown that an increasing Genetic Risk Score, or number of risk alleles, is associated with decreased serum 25(OH)D levels or vitamin D deficiency/insufficiency using the candidate SNPs (Engelman et al. 2013; Lu et al. 2012; Signorello et al. 2011; Zhang et al. 2013). Each study used a different set of SNPs, and the strongest signals of association were found at different SNPs. There is heterogeneity in the study of SNP allele frequencies and LD variation across the studied populations. Without knowing the causal variants, such analyses may provide a limited interpretive value for prevention strategies or recommendations for vitamin D supplementation.

Differences in behavioral and environmental factors, variation in LD across different populations, and the small sample size of this study likely caused the inconsistencies observed for associations across studies. Our EA study participants were more likely to have higher education levels and income, and more prevalent use of vitamin D supplementation than AA study participants (Murphy et al. 2012). Moreover, many believe that consistently significant differences in serum 25(OH)D levels between AAs and EAs even after adjusting for relevant behavioral, biological, and environmental variables suggest that skin pigmentation is one of the major contributing factors (Harris 2011; Harris and Dawson-Hughes 1998; Signorello et al. 2010). However, the impact of skin pigmentation differences between AAs and EAs on the observed difference in serum vitamin D levels and contrasting pattern of associations is still not clear. In this project, we used an objective method of measuring skin pigmentation and skin pigmentation was not associated with serum vitamin D levels in AAs. Our AA subjects exhibited large variation in skin color and thus more analyses are necessary to understand the role of skin pigmentation on vitamin D disparities.

The limitations of this study are the low number of tagging SNPs genotyped and small sample size. We selected a small number of tagging SNPs that are most likely in LD with causal variants, but the tagging SNPs selected based on the GWAS in European populations may not be suitable in AAs. Fine mapping around the region with strong signals of association in our AAs may help identify other variants that show stronger association and find functional variants that may affect vitamin D synthesis and metabolism. Compared to the previous GWAS meta-analyses (Ahn et al. 2010; Wang et al. 2010), our study had small sample size. Despite small sample size, our EA samples size was sufficient to observe the significant associations for the GWAS top hint in *GC* and *CYP2R1*. The genetic effect in AAs, on the other hand, was smaller, and we may not have had enough statistical power. This study, however, is the second largest that examined association of circulating 25(OH)D levels and genetic variations in AA populations. This study also incorporated many biological and environmental modifiers of serum 25(OH)D levels, including objective measurements of skin pigmentation, into the analyses. Combined effect of gene and environment likely explains the difference in prevalence of vitamin D deficiency between AAs and EAs (Moonesinghe et al. 2012). Our future studies will explore whether gene–environment interactions play a role in vitamin D disparities between AAs and EAs.

Vitamin D deficiency is very common among AAs and is considered to be a potential contributor to health disparities. For EA adults, increasing vitamin D intake from supplementation or diet to more than 600 IU/day is necessary to keep serum 25(OH)D levels at 50 nmol/l (20 ng/ml), but maintaining levels ≥75 nmol/l (30 ng/ml) likely requires more than 1,500 IU/day of vitamin D (Holick et al. 2011; Institute of Medicine 2011; Ross et al. 2011). Administering 2,000 IU/day of vitamin D supplementation is shown to raise serum vitamin D to sufficient levels in AA populations (Dong et al. 2010; Talwar et al. 2007), and 4,000 IU/day of vitamin D supplement intake eliminated the differences in serum 25(OH)D levels between AA and EA men (Garrett-Mayer et al. 2012). These studies, however, observed a considerable variation in serum 25(OH)D levels, even after accounting for vitamin D supplement intake. Another study investigating the impact of vitamin D supplementation also observed high variation in serum 25(OH)D levels within each category of vitamin D supplement dose (Garland et al. 2011). UVR exposure, skin color, season, BMI, and other environmental factors contribute to the variation, but biological factors and genetic variation in vitamin D pathway genes affecting rate of 25-hydroxylation likely play a role in dose response to vitamin D supplementation (McDonnell et al. 2013; Nimitphong et al. 2013). For instance, AA individuals who carry genetic variants that reduce vitamin D hydroxylation and binding protein stability may require substantially higher doses of vitamin D supplementation to maintain optimal serum 25(OH)D levels.

In summary, this study successfully replicated several GWAS-identified SNPs associated with 25(OH)D levels in AA and EA populations. The results also provide evidence that *CYP2R1* and *GC* contribute more to serum 25(OH)D variation than other genes in the vitamin D pathway in our study populations. The contrasting pattern of associations between AAs and EAs suggests that additional studies are warranted to identify the causal variants affecting vitamin D binding and enzymatic activities.

## Electronic supplementary material

Below is the link to the electronic supplementary material.

Supplementary material 1 (PDF 431 kb)
