# Supplementary material for: Common vitamin D pathway gene variants reveal contrasting effects on serum vitamin D levels in African Americans and European Americans
Source: Hum Genet. 2014 Aug 2;133(11):1395–405. doi: 10.1007/s00439-014-1472-y (PMC4185105; doi:10.1007/s00439-014-1472-y)
Supplement: Supplementary file 1 — Supplementary material 1 (PDF 431 kb) [file 439_2014_1472_MOESM1_ESM.pdf]

Supplementary Materials

Supplementary Table 1 SNPs analyzed in this study

Supplementary Table 2 Results of linear regression analysis of biological and environmental modifiers of serum 25(OH)D levels

Supplementary Table 3 Results of linear regression analysis in African Americans

Supplementary Table 4 Results of linear regression analysis in European Americans

Supplementary Table 5 Summary of Genome Wide Association Studies identified variants association tests (*P*-values)

Supplemental Figure 1 Distribution of serum 25(OH)D levels in African Americans and European Americans showing higher prevalence of vitamin D deficiency in African Americans compared to European Americans

Supplemental Figure 2 LD plots of three gene regions (*GC*, *CYP2R1*, and *DHCR7/NADSYN1*) in AAs and EAs

References

Supplementary Table 1 SNPs analyzed in this study

Chromosome	SNP	Position (BP)[a]	Genes	References
2	rs116071925	219646701	*CYP27A1*	
4	rs17467825	72605517	*GC*	(Wang et al. 2010)
	rs2282679	72608383	*GC*	(Wang et al. 2010)
	rs3755967	72609398	*GC*	(Wang et al. 2010)
	rs2298850	72614267	*GC*	(Wang et al. 2010)
	rs7041	72618334	*GC*	(Ahn et al. 2010; Wang et al. 2010)
	rs1155563	72643488	*GC*	(Ahn et al. 2010; Wang et al. 2010)
	rs3733359	72649774	*GC*	
	rs16847024	72650679	*GC*	
	rs115316390	72651159	*GC*	
7	rs2740574	99382096	*CYP3A4*	
11	rs1993116	14910234	*CYP2R1*	(Ahn et al. 2010; Wang et al. 2010)
	rs12794714	14913575	*CYP2R1*	(Wang et al. 2010)
	rs114050796	14914653	*CYP2R1*	
	rs10741657	14914878	*CYP2R1*	(Wang et al. 2010)
	rs2060793	14915310	*CYP2R1*	(Ahn et al. 2010; Wang et al. 2010)
	rs7944926	71165625	*DHCR7/NADSYN1*	(Wang et al. 2010)
	rs12785878	71167449	*DHCR7/NADSYN1*	(Wang et al. 2010)
	rs4944957	71168035	*DHCR7/NADSYN1*	(Wang et al. 2010)
	rs12800438	71171003	*DHCR7/NADSYN1*	(Wang et al. 2010)
	rs3794060	71187679	*DHCR7/NADSYN1*	(Wang et al. 2010)
	rs3829251	71194559	*DHCR7/NADSYN1*	(Ahn et al. 2010)
	rs4945008	71221248	*DHCR7/NADSYN1*	(Wang et al. 2010)
	rs11234027	71234107	*DHCR7/NADSYN1*	(Ahn et al. 2010)
12	rs11574143	48234917	*VDR*	
	rs731236 (*Taq*I)	48238757	*VDR*	
	rs1544410 (*Bsm*I)	48239835	*VDR*	
	rs2228570 (*Fok*I)	48272895	*VDR*	

	rs11574038	48277153	*VDR*	
	rs1989969	48278010	*VDR*	
	rs11568820 (*Cdx*2)	48302545	*VDR*	
	rs4646537	58157281	*CYP27B1*	
	rs10877012	58162085	*CYP27B1*	
20	rs6013897	52742479	*CYP24A1*	(Wang et al. 2010)
	rs6022990	52775532	*CYP24A1*	
	rs73913755	52790194	*CYP24A1*	
	rs73913757	52790518	*CYP24A1*	
	rs2248359	52791518	*CYP24A1*	
	rs2248461	52792202	*CYP24A1*	

[a] Base pair position on the chromosome is based on GRCh37/hg19

Supplementary Table 2 Results of linear regression analysis of biological and environmental modifiers of serum 25(OH)D levels

	African Americans		European Americans	
	β	*P*	*β*	*P*
Age	0.000	0.86	0.003	0.006
Total Vitamin D Intake (≥400 IU/day vs <400 IU/day)	0.165	<0.001	0.160	<0.001
Season of Blood Draw (UV high seasons vs. low season)	0.117	<0.001	0.066	<0.001
Study Site (Chicago vs. Washington, D.C.)	0.110	<0.001	NA	NA
UVR Exposure (high vs. medium and low exposure	0.031	0.21	0.046	0.06
Body Mass Index	0.001	0.09	-0.007	<0.001
M-Index	-0.002	0.14	0.000	0.95
Education (≤high school vs. > high school)	0.012	0.54	0.023	0.32
West African Ancestry	0.005	0.96	NA	NA
European Ancestry	NA	NA	0.049	0.67

Supplementary Table 3 Results of linear regression analysis in African Americans

				Model 1[a] (n=652)				Model 2[b] (n=557)			
Chromosome	SNPs	MA[d]	MAF[e]	*β*	*P*	EMP1[f]	EMP2[g]	*β*	*P*	EMP1[f]	EMP2[g]
2	rs116071925	A	0.017	-0.03	0.59	0.59	1.00	-0.04	0.34	0.34	0.99
4	rs17467825	G	0.111	-0.02	0.25			-0.01	0.62		
	rs2282679	C	0.102	-0.03	0.13			-0.01	0.68		
	rs3755967	A	0.110	-0.04	0.06			-0.02	0.30		
	rs2298850	G	0.090	-0.04	0.06			-0.01	0.65		
	rs7041	G	0.189	0.00	0.81			0.01	0.57		
	rs1155563	C	0.091	-0.04	0.09			-0.04	**0.048**		
	rs3733359	T	0.238	-0.01	0.33	0.33	1.00	-0.01	0.29	0.29	0.99
	rs16847024	T	0.085	-0.02	0.43	0.42	1.00	-0.01	0.54	0.54	1.00
	rs115316390	A	0.007	0.09	0.25	0.24	0.99	0.17	**0.03**	**0.03**	0.33
7	rs2740574	A	0.403	-0.01	0.40	0.40	1.00	-0.01	0.68	0.68	1.00
11	rs1993116	T	0.276	0.02	0.08			0.03	**0.02**		
	rs12794714	A	0.161	-0.03	0.05			-0.04	**0.01**		
	rs114050796	T	0.062	0.01	0.57	0.40	1.00	0.03	0.20	0.19	0.96
	rs10741657	A	0.273	0.03	**0.04**			0.04	**0.01**		
	rs2060793	A	0.358	0.03	0.07			0.03	**0.02**		
	rs7944926	G	0.267	0.00	0.88			-0.02	0.25		
	rs12785878	T	0.271	0.00	0.90			-0.02	0.26		
	rs4944957	G	0.493	0.03	**0.02**			0.02	0.21		
	rs12800438	A	0.392	0.04	**0.002**			0.02	0.07		
	rs3794060	T	0.245	0.00	0.82			-0.01	0.60		
	rs3829251	A	0.230	-0.01	0.45			0.00	0.89		
	rs4945008	G	0.245	0.00	0.83			-0.01	0.65		
	rs11234027	A	0.290	0.01	0.36			0.02	0.12		
12	rs11574143	A	0.092	-0.02	0.30	0.30	1.00	-0.02	0.43	0.45	1.00
	rs731236	C	0.277	-0.02	0.09	0.09	0.77	-0.02	0.14	0.14	0.88
	rs1544410	A	0.285	-0.02	0.24	0.24	0.99	-0.02	0.16	0.16	0.92

	rs11574038	A	0.032	-0.01	0.79	0.78	1.00	-0.01	0.88	0.87	1.00
	rs4646537	C	0.088	0.02	0.33	0.33	1.00	0.01	0.77	0.76	1.00
	rs10877012	T	0.145	-0.03	0.11	0.11	0.82	-0.01	0.53	0.52	1.00
20	rs6013897	A	0.259	0.00	0.96			0.00	0.87		
	rs6022990	G	0.086	0.00	0.93	0.93	1.00	0.03	0.13	0.14	0.88
	rs73913755	A	0.179	0.00	0.81	0.81	1.00	0.01	0.56	0.56	1.00
	rs73913757	T	0.145	0.00	0.99	0.99	1.00	0.00	0.99	0.99	1.00
	rs2248359	C	0.362	-0.02	0.18	0.18	0.96	-0.02	0.22	0.21	0.97
	rs2248461	G	0.366	-0.01	0.39	0.39	1.00	-0.01	0.43	0.42	1.00

[a] Adjusted for age, WAA, and study site
[b] Adjusted for age, WAA, study site, total vitamin D intake, and season of blood draw
[c] Base pair position on the chromosome is based on GRCh37/hg19
[d] Minor Allele
[e] Minor Allele Frequency
[f] Pointwise empirical *P*-values obtained using max(T) permutation procedure (10,000 permutations)
[g] Empirical *P*-values correcting for multiple testing

Supplementary Table 4 Results of linear regression analysis in European Americans

				Model 1[a] (n=405)				Model 2[b] (n=385)			
Chromosome	SNPs	MA[d]	MAF[e]	*β*	*P*	EMP1[f]	EMP2[g]	*β*	*P*	EMP1[f]	EMP2[f]
4	rs17467825	G	0.266	-0.05	**0.004**			-0.04	**0.003**		
	rs2282679	C	0.265	-0.05	**0.0014**			-0.05	**0.0014**		
	rs3755967	A	0.268	-0.04	**0.005**			-0.04	**0.002**		
	rs2298850	G	0.244	-0.03	0.08			-0.03	**0.039**		
	rs7041	T	0.418	-0.04	**0.009**			-0.04	**0.0007**		
	rs1155563	C	0.206	-0.03	0.13			-0.02	0.31		
	rs3733359	T	0.077	0.04	0.11	0.12	0.99	0.01	0.80	0.80	1.00
	rs16847024	T	0.007	-0.02	0.75	0.75	1.00	-0.03	0.60	0.58	1.00
	rs115316390	A	0.005	-0.02	0.87	0.87	1.00	0.07	0.44	0.44	1.00
7	rs2740574	G	0.064	-0.04	0.11	0.10	0.99	-0.03	0.26	0.25	1.00
11	rs1993116	T	0.382	0.05	**0.0005**			0.04	**0.0006**		
	rs12794714	A	0.432	-0.04	**0.004**			-0.04	**0.005**		
	rs114050796	T	0.014	-0.03	0.62	0.62	1.00	-0.01	0.86	0.86	1.00
	rs10741657	A	0.375	0.05	**0.002**			0.04	**0.003**		
	rs2060793	A	0.385	0.04	**0.002**			0.04	**0.005**		
	rs7944926	A	0.301	-0.02	0.22			-0.01	0.45		
	rs12785878	G	0.297	-0.02	0.20			-0.01	0.51		
	rs4944957	A	0.283	-0.02	0.26			-0.01	0.52		
	rs12800438	G	0.293	-0.02	0.26			-0.01	0.53		
	rs3794060	C	0.299	-0.02	0.27			-0.01	0.53		
	rs3829251	A	0.149	0.00	0.95			0.02	0.29		
	rs4945008	A	0.300	-0.02	0.23			-0.01	0.46		
	rs11234027	A	0.150	0.00	0.97			0.02	0.34		
12	rs11574143	A	0.111	0.03	0.17	0.17	0.99	0.01	0.49	0.50	1.00
	rs731236	C	0.384	0.00	0.98	0.98	1.00	0.00	0.75	0.75	1.00
	rs1544410	A	0.369	0.00	0.89	0.88	1.00	0.01	0.46	0.46	1.00
	rs11574038	A	0.002	0.13	0.36	0.34	0.99	0.03	0.80	0.80	1.00

	rs11568820	A	0.248	-0.03	0.13	0.14	0.84	-0.02	0.25	0.25	0.98
	rs4646537	C	0.069	0.00	0.86	0.86	1.00	-0.03	0.32	0.32	1.00
	rs10877012	T	0.316	0.00	0.92	0.92	1.00	0.00	0.93	0.93	1.00
20	rs6013897	A	0.201	0.00	0.98			0.00	0.75		
	rs6022990	G	0.011	0.08	0.25	0.25	0.97	0.03	0.60	0.59	1.00
	rs73913755	A	0.067	-0.01	0.68	0.68	1.00	-0.03	0.20	0.20	0.97
	rs73913757	T	0.014	-0.11	**0.04**	**0.04**	0.47	-0.09	**0.04**	**0.04**	0.49
	rs2248359	T	0.435	0.01	0.55	0.55	1.00	0.00	0.79	0.79	1.00
	rs2248461	A	0.420	0.01	0.61	0.60	1.00	0.00	0.89	0.88	1.00

[a] Adjusted for age
[b] Adjusted for age, total vitamin D intake, and season of blood draw, BMI, and UV exposure
[c] Base pair position on the chromosome is based on GRCh37/hg19
[d] Minor Allele
[e] Minor Allele Frequency
[f] Pointwise empirical *P*-values obtained using max(T) permutation procedure (10,000 permutations)
[g] Empirical *P*-values correcting for multiple testing

Supplementary Table 5 Summary of genome wide association studies identified variants association tests (*P*-values)

Gene	SNPs	MA in CEU[a]	Wang et al. 2010	Ahn et al. 2010	EAs in this study[b]		AAs in this study[c]		MA in YRI[d]
					β	*P*	*β*	*P*	
GC	rs17467825	G	6.8×10^{-74}		-0.04	**0.003**	-0.01	0.62	G
	rs2282679	C	1.9×10^{-109}	1.8×10^{-49}	-0.05	**0.0014**	-0.01	0.68	C
	rs3755967	A	2.4×10^{-75}		-0.04	**0.002**	-0.02	0.30	A
	rs2298850	G	2.0×10^{-71}		-0.03	**0.04**	-0.01	0.65	G
	rs7041	T	6.3×10^{-59}		-0.02	**0.0007**	0.01	0.57	T
	rs1155563	C	2.4×10^{-73}		-0.02	0.31	-0.04	**0.048**	C
CYP2R1	rs1993116	A	6.3×10^{-11}	2.9×10^{-17}	0.04	**0.0006**	0.03	**0.02**	A
	rs12794714	A	2.7×10^{-9}		-0.04	**0.005**	-0.4	**0.01**	A
	rs10741657	A	3.3×10^{-20}		0.04	**0.003**	0.04	**0.01**	A
	rs2060793	A	1.7×10^{-11}	1.4×10^{-5}	0.04	**0.005**	0.03	**0.02**	A
DHCR7/NADSYN1	rs7944926	A	9.0×10^{-16}		-0.01	0.45	-0.02	0.25	G
	rs12785878	G	2.1×10^{-27}		-0.01	0.51	-0.02	0.26	T
	rs4944957	A	8.7×10^{-15}		-0.01	0.52	0.02	0.21	G
	rs12800438	G	2.5×10^{-15}		-0.01	0.53	0.02	**0.07**[e]	A
	rs3794060	C	3.4×10^{-15}		-0.01	0.53	-0.01	0.60	T
	rs3829251	A		8.8×10^{-7}	0.02	0.29	0.00	0.89	A
	rs4945008	A	4.6×10^{-15}		-0.01	0.46	-0.01	0.65	G
	rs11234027	A		3.4×10^{-9}	0.02	0.34	0.02	0.12	A
CYP24A1	rs6013897	A	6.0×10^{-10}		0.00	0.75	0.00	0.87	A

[a] Minor Allele Frequency in 1000 Genomes Project CEU (Phase I May 2011 release)

[b] Linear regression adjusted for age, total vitamin D intake, and season of blood draw, BMI, and UVR exposure (Model 2)

[c] Linear regression adjusted for age, WAA, study site, total vitamin D intake, and season of blood draw (Model 2)

[d] Minor Allele Frequency in 1000 Genomes Project YRI (Phase I May 2011 release)

[e] rs12800438 was significantly associated with vitamin D deficiency (*P*=0.04) in logistic regression analysis adjusted for age, WAA, study site, total vitamin D intake, and season of blood draw.

Supplemental Figure 1 Distribution of serum 25(OH)D levels in African Americans and European Americans showing higher prevalence of vitamin D deficiency in African Americans compared to European Americans

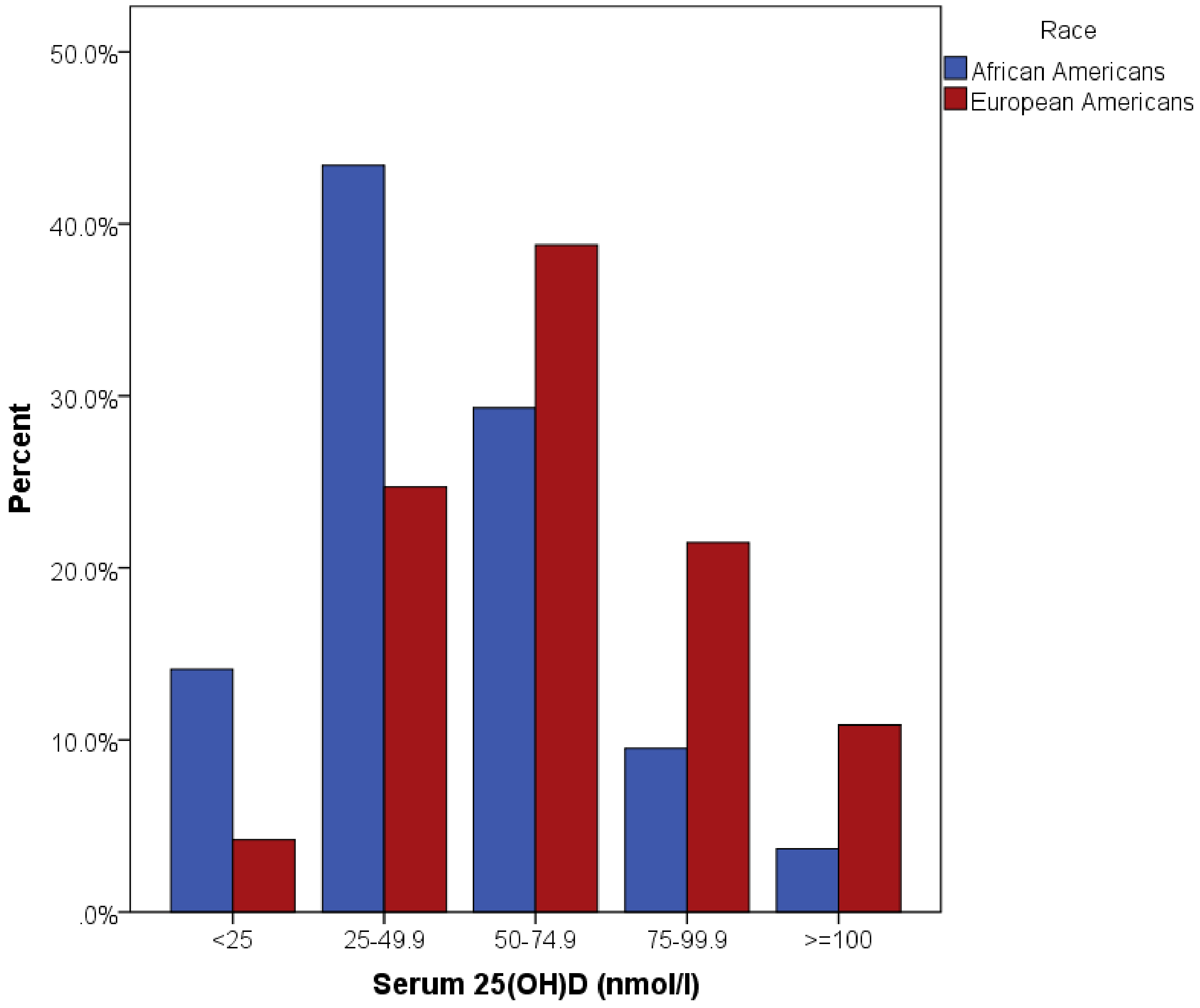

Supplemental Figure 2 LD plots of three gene regions (*GC*, *CYP2R1*, and *DHCR7/NADSYN1*) in AAs and EAs

AAs *GC*

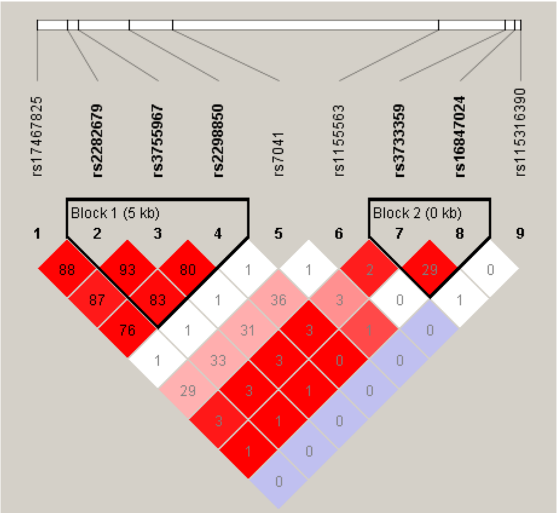

CYP2R1

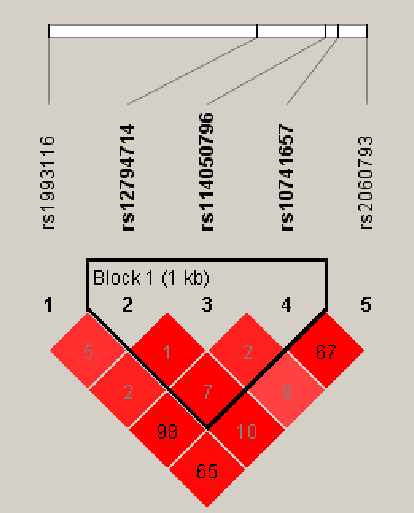

DHCR7/NADSYN1

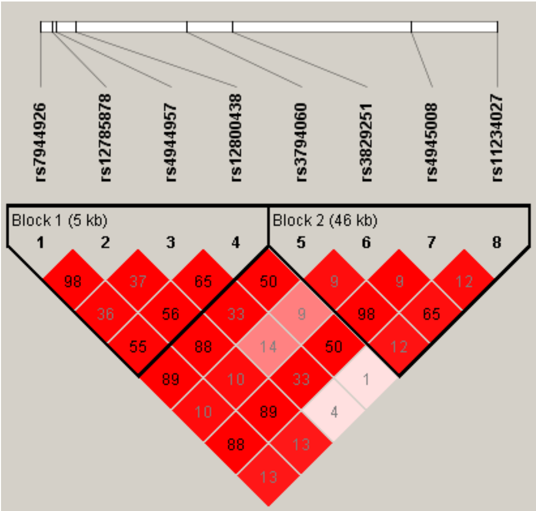

EAs *GC*

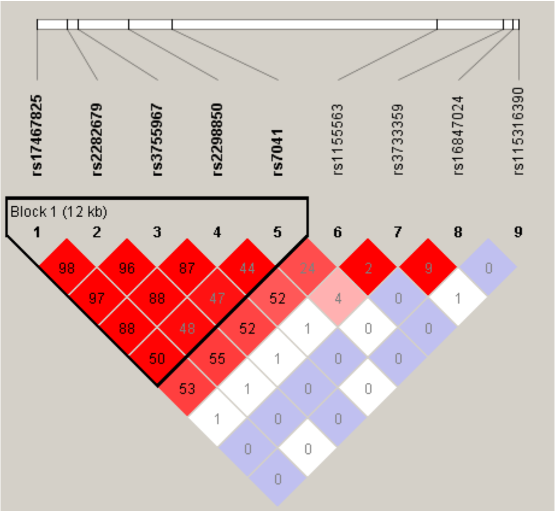

CYP2R1

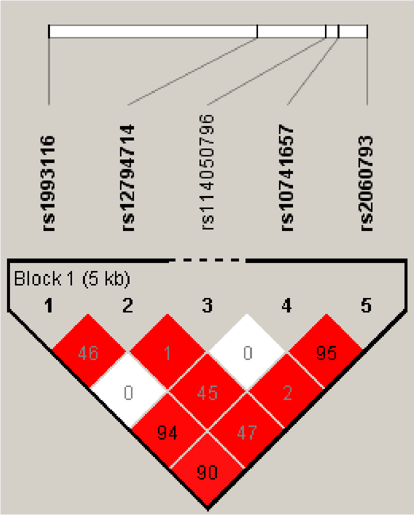

DHCR7/NADSYN1

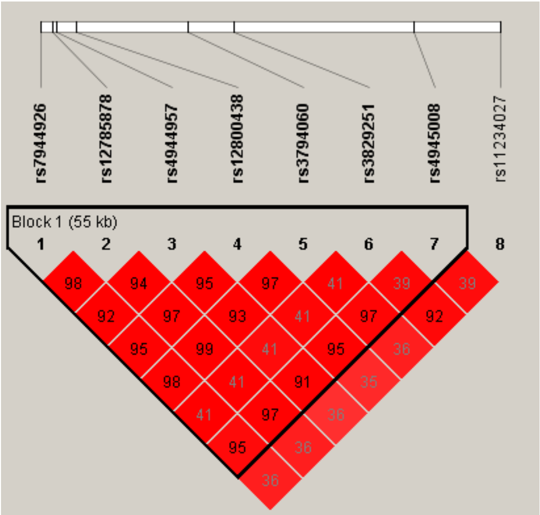

References

Ahn J, Yu K, Stolzenberg-Solomon R, Simon KC, McCullough ML, Gallicchio L, Jacobs EJ, Ascherio A, Helzlsouer K, Jacobs KB, Li Q, Weinstein SJ, Purdue M, Virtamo J, Horst R, Wheeler W, Chanock S, Hunter DJ, Hayes RB, Kraft P, Albanes D (2010) Genome-wide association study of circulating vitamin D levels. Hum Mol Genet 19: 2739-2745.

Wang TJ, Zhang F, Richards JB, Kestenbaum B, van Meurs JB, Berry D, Kiel DP, Streeten EA, Ohlsson C, Koller DL, Peltonen L, Cooper JD, O'Reilly PF, Houston DK, Glazer NL, Vandenput L, Peacock M, Shi J, Rivadeneira F, McCarthy MI, Anneli P, de Boer IH, Mangino M, Kato B, Smyth DJ, Booth SL, Jacques PF, Burke GL, Goodarzi M, Cheung C-L, Wolf M, Rice K, Goltzman D, Hidiroglou N, Ladouceur M, Wareham NJ, Hocking LJ, Hart D, Arden NK, Cooper C, Malik S, Fraser WD, Hartikainen A-L, Zhai G, Macdonald HM, Forouhi NG, Loos RJF, Reid DM, Hakim A, Dennison E, Liu Y, Power C, Stevens HE, Jaana L, Vasan RS, Soranzo N, Bojunga J, Psaty BM, Lorentzon M, Foroud T, Harris TB, Hofman A, Jansson J-O, Cauley JA, Uitterlinden AG, Gibson Q, Järvelin M-R, Karasik D, Siscovick DS, Econs MJ, Kritchevsky SB, Florez JC, Todd JA, Dupuis J, Hyppönen E, Spector TD (2010) Common genetic determinants of vitamin D insufficiency: a genome-wide association study. Lancet 376: 180-188.
